# The Importance of the Pathological Perspective in the Management of the Invasive Lobular Carcinoma

**DOI:** 10.1155/2022/2461242

**Published:** 2022-09-26

**Authors:** Funda Tasli, Demet Cavdar, Sibel Demir Kececi, Baha Zengel, Zehra Hilal Adibelli, Gamze Dal, Irem Gonen, Ozden Oz, Cengiz Yilmaz, Ozlem Ozdemir, Hulya Mollamehmetoglu, Ismail Dilek, Enver Ilhan, Adam Uslu

**Affiliations:** ^1^Health Sciences University, Izmir Faculty of Medicine, Department of Pathology, Izmir Faculty of Medicine Bozyaka Training Hospital, Izmir, Turkey; ^2^Manisa Merkez Efendi Hospital, Department of Pathology, Manisa Merkez Efendi State Hospital, Manisa, Turkey; ^3^Igdir State Hospital, Department of Radiology, Pir Sultan Abdal, Melekli Street, Igdır, Turkey

## Abstract

**Background:**

Invasive lobular carcinomas (ILC) account for 10–15% of all breast cancers and are the second most common histological form of breast cancer. They usually show a discohesive pattern of single cell infiltration, tend to be multifocal, and the tumor may not be accompanied by a stromal reaction. Because of these histological features, which are not common in other breast tumors, radiological detection of the tumor may be difficult, and its pathological evaluation in terms of size and spread is often problematic. The SSO-ASTRO guideline defines the negative surgical margin in breast-conserving surgeries as the absence of tumor detection on the ink. However, surgical margin assessment in invasive lobular carcinomas has not been much discussed from the pathological perspective.

**Methods:**

The study included 79 cases diagnosed with invasive lobular carcinoma by a Tru-cut biopsy where operated in our center between 2014 and 2021. Clinicopathological characteristics of the cases, results of an intraoperative frozen evaluation in cases that underwent conservative surgery, the necessity of re-excision and complementary mastectomy, and consistency in radiological and pathological response evaluation in cases receiving neoadjuvant treatment were questioned.

**Results:**

The tumor was multifocal in 37 (46.8%) cases and single tumor focus in 42 (53.2%) cases. When the entire patient population was evaluated, regardless of focality, mastectomy was performed in 27 patients (34.2%) and breast-conserving surgery (BCS) was performed in 52 patients (65.8%). Of the 52 patients who underwent BCS, 26 (50%) required an additional surgical procedure (cavity revision or completion mastectomy). There is a statistical relationship between tumor size and additional surgical intervention (*p* < 0.05). BCS was performed in 7 of 12 patients who were operated on after neoadjuvant treatment, but all of them were reoperated with the same or a second session and turned to mastectomy. Neoadjuvant treatment and the need for reoperation were statistically significant (*p* < 0.05). Additional surgical procedures were performed in 20 (44.4%) of 45 patients in BCS cases who did not receive neoadjuvant therapy.

**Conclusions:**

Diagnostic difficulties in the intraoperative frozen evaluation of invasive lobular carcinoma are due to the different histopathological patterns of the ILC. In our study, it was determined that large tumor size and neoadjuvant therapy increased the need for additional surgical procedures. It is thought that the pathological perspective is the determining factor in order to minimize the negative effects such as unsuccessful cosmesis, an additional surgical burden on the patient, and cost increase that may occur with additional surgical procedures; for this reason, new approaches should be discussed in the treatment planning of invasive lobular carcinoma cases.

## 1. Introduction

Invasive lobular carcinomas (ILC) account for 10–15% of all breast cancers and are the second most common histological form of breast cancer. Classical ILC is characterized by the characteristic single-row formation of discohesive cells that infiltrate the breast stroma without producing desmoplastic stroma. Cells are often dispersed separately along a fibrous connective tissue or organized into single-row linear cords that invade the fibrous stroma [1–3]. The developmental pattern of classical single cell infiltrates in the ILC and the lack of stromal reactions make it difficult to determine the size of the tumor and its boundaries within the breast parenchyma [4]. Mostly, the immune response to the tumor is minimal. ILC is often associated with lobular carcinoma in situ. It is now recognized as a biologically distinct disease from the more common invasive ductal carcinoma [1–3].

Intraoperative resection margin assessment is widely used in the breast-conserving surgery (BCS) procedure, as it significantly reduces reoperation rates, and many techniques have been developed for this [5]. The techniques frequently reported in the literature are frozen section analysis and imprint cytology. The frozen section is the most commonly used method in the BCS procedure. In this technique, the sample is frozen and sectioned, followed by microscopic examination by thawing, fixing, and staining [6]. Tubular carcinoma and ILC are reported to be the most common mammary tumors that may have diagnostic difficulties in frozen section examination. Freezing and microscopic examination of all borders may cause erroneous results, especially due to difficulties in freezing and sectioning of adipose tissue. In frozen evaluation; Factors such as freezing and thawing artifact, irregular surface of the breast tissue, and thermal damage of the breast parenchyma caused by electrocautery, especially at surgical margins, may complicate the evaluation of surgical margins in frozen for some tumors such as ILC [7].

Safe surgical margins for the BCS procedure are defined in the 2014 Society of Surgical Oncology (SSO)-American Society for Radiation Oncology (ASTRO) guidelines as “no tumor cells in the ink” for all invasive breast carcinomas, including the invasive lobular subtype. Retrospective studies that provided guidelines for the lobular subtype examined tumor recurrence, disease-free, and overall survival in the same breast, and reported similar results in ductal and lobular carcinomas [8–11]. However, in these studies, the lobular subtype is approximately 10% of the patient population, and the necessity and frequency of reoperation have not been discussed.

In this study, we aimed to investigate how the pathological evaluation in the frozen session shapes the surgical procedure, the results of the surgical approach, and the difficulties and pitfalls in the practice of pathology in patients with a diagnosis of ILC who underwent BCS.

## 2. Materials and Methods

### 2.1. Study Population

The study included 79 cases diagnosed with invasive lobular carcinoma by a Tru-cut biopsy and operated in our center between 2014 and 2021. The radiological findings of the cases, the surgical procedures performed, age, tumor size, lymph node status, multifocality, presence of in situ lesions, molecular subtype, neoadjuvant status (yes/no), type of surgery (breast-conserving surgery/complementary mastectomy/mastectomy), conservative surgery performed cavity revision request in frozen session in cases, presence of tumor in revision (presence/absent), compatibility in radiological and pathological treatment response evaluation in cases who received neoadjuvant treatment were examined.

### 2.2. Pathological Examination

In the BCS procedure, the lumpectomy materials sent to the pathology laboratory for intraoperative pathological analysis, oriented with sutures, were first positioned according to the sutures and evaluated considering 6 surgical margins (anterior, posterior, lateral, medial, superior, and inferior). The outer surface of the material is painted with ink for surgical margins. After the ink was dried, 5 mm thick sections were made on the material in the anterior-posterior plane. The dimensions of the observed tumor and its distance from the surgical margins were determined macroscopically. Samples were taken for microscopic examination from the nearest surgical margins and fibrotic areas at the surgical margins in palpable tumor-free materials.

After the samples were frozen in the frozen device, 5 micron thick sections were obtained, and they were taken on the preparations and stained with hematoxylin and eosin staining. Stained preparations were evaluated by 2 expert pathologists. Although the duration of the frozen procedure varies only when accompanied by surgical margin evaluation or sentinel sampling, it is around 15 minutes on average. The 2014 ASCO/ASTRO guideline was based on the evaluation of surgical margin positivity.

### 2.3. Statistical Analysis

Statistical analyzes were performed using the SPSS 21.0 software for Windows. All statistical tests were two-sided, and a *p* value < 0.05 was considered statistically significant.

Quantitative variables were compared by Pearson's chi-square test. Qualitative variables were compared by the variance analysis (ANOVA). For non-normally distributed variables, a nonparametric analysis was performed (Mann–Whitney *U* test). A logistic regression model was used for multivariate analysis.

## 3. Result

All 79 cases were female. The youngest age was 36, the highest was 82 and the mean age was 59 and 48 years. Twenty (25.3%) of the cases are under the age of 50. The clinical and biological characteristics of the cases are summarized in Table 1. When the entire patient population was evaluated; mastectomy was performed in 27 patients (34.2%) and breast-conserving surgery (BCS) was performed in 52 patients (65.8%). In the cases that underwent mastectomy and BCS; there is statistical significance with the presence of in situ component and tumor size (*p* < 0.05).

Cavity revision was performed in 8 (15.4%) of 52 patients who underwent BCS, and completion mastectomy was performed in 18 (34.6%) patients. In other words, an additional surgical procedure was performed for safe surgical margins in 26 (50%) of the patients who underwent BCS. The need for additional surgical procedures in BCS cases was evaluated with multifocality, tumor size, presence of in situ component, lymph node metastasis, and neoadjuvant treatment (Table 2). Additional surgery was found to be statistically significant in those receiving neoadjuvant treatment (*p* < 0.05). There was also statistical significance between tumor size and additional surgery (*p* < 0.05). The presence of in situ components, lymph node status, and multifocality are unrelated to additional surgery.

No tumor was detected in the reoperation materials in 12 (46.1%) of 26 patients who underwent cavity revision or completion mastectomy. There is statistical significance between the presence of tumor and tumor size in the reoperation materials (*p* < 0.05). Multifocality, neoadjuvant therapy, lymph node status, and presence of in situ components are not associated with the presence of residual tumor in additional surgical material.

There are 12 patients in our series who received neoadjuvant therapy. No pathological complete response was observed in any patient, and 9 (75%) had no response and 3 (25%) had a partial response. When radiological response evaluation and pathological response were compared after neoadjuvant therapy; All 7 cases, which were evaluated as radiological complete-near complete response, were pathologically unresponsive. Additional surgical procedure (complementary mastectomy or cavity revision) was performed in 6 (85.7%) of 7 patients who underwent BCS. Neoadjuvant therapy uptake and surgical procedures performed in the entire patient population are summarized in Table 3.

## 4. Discussion

BCS is defined as the removal of the tumor along with the surrounding normal breast tissue without impairing the cosmetic appearance of the breast [12]. It is the primary treatment for early-stage breast cancer. The most commonly used method for the evaluation of intraoperative surgical margins is the “frozen section” method [5]. Nowikiewicz et al. reported that the intraoperative frozen evaluation method reduces the frequency of reoperation in lumpectomy patients. But only 8% of the study population has ILC patients and the frequency of reoperation according to histological subtypes has not been reported [6]. Additional surgical procedures in ILC are reported in the literature at a rate of 23.1%–44.5% [2, 13]. In their study, Hewit et al. reported that 10.6% of ILC patients who underwent total mastectomy had positive surgical margins and that surgical margin positivity was associated with large tumor size and short recurrence-free survival [14].

**Figure 1:**
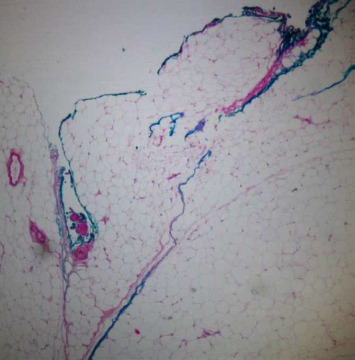
Penetration of ink between oil lobules (x40; H*δ*E).

**Figure 2:**
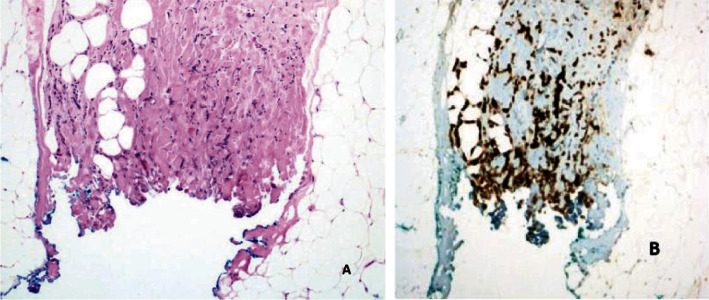
(a) Inability to clearly assess cellular detail at surgical margins due to electrocautery artifact (x100, HE). (b) Demonstration of tumor cells by IHC method in permanent sections (x100, IHC Cytokeratin).

**Figure 3:**
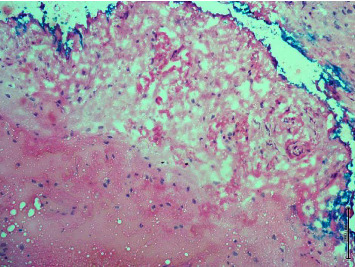
Possible tumoral cells similar to lymphocyte or stromal cells (Frozen Section, x200, H*δ*E).

**Figure 4:**
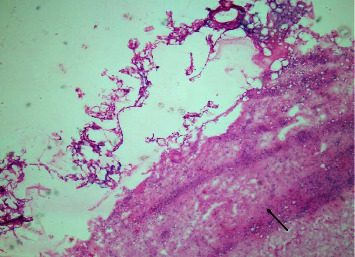
Freezing artifact: separation of adipose tissue and the painted surgical margin from fibrous tissue (arrow: fibrous tissue) (Frozen Section, x40; H*δ*E).

**Figure 5:**
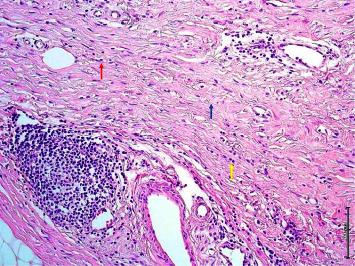
Stromal cells (blue arrow), tumor cells (yellow arrow), and lymphocytes (red arrow) on a fibrous background after neoadjuvant therapy (x200; H*δ*E).

In our study, surgical margin positivity was not found in cases with primary mastectomy. All 52 patients who underwent BCS were evaluated with the intraoperative frozen section in terms of surgical margins, and 26 (50%) of 52 patients underwent additional surgical procedures due to positive surgical margins. There is a relationship between large tumor size and additional surgical procedures. The high rate of need for additional surgery in our study, which is similar to the literature, may be related to the fact that tumors are not suitable for frozen evaluation due to the different histological patterns of the tumor. If we discuss the ILC surgical margin evaluation from a pathological point of view (1) frozen surgical margin assessment begins with the identification of the invasive margins of the tumor by palpation and macroscopic examination. The pathological response to invasion is the disorganized arrangement of tumor cells with a desmoplastic reaction that gives the tumor its stiffness. Tumors cannot be localized by palpation because the stromal reaction is not usually expected in ILC. Since the tumor spreads along the fibrotic bands of the usual breast parenchyma, it is often not confined and it is difficult to determine the sampling site for microscopic examination. (2) BCS materials do not have a smooth surface as the parenchyma consists of adipose tissue. The penetration of the ink applied for the surgical margin into the fat lobules may make it difficult to determine the true resection margin (Figure 1). (3) Electrocautery used during surgery causes thermal burns in the tissue. This artifact that occurs at the surgical margins disrupts the structural and cytological details of the cells (Figure 2). The hypocellular nature of the tumor and the quiescent nuclear features seen in ILC may cause the tumor cells to mix with lymphocytes dispersed as a single cell (Figure 3). (4) Frozen process damages the tissue by freezing and thawing processes, especially in fatty tissues, this damage can result in tissue loss and failure in sectioning (Figure 4). In addition, it is statistically significant to apply more additional surgical procedures to those receiving neoadjuvant therapy in our study. After neoadjuvant therapy, fibrosis occurs in the surrounding breast parenchyma as well as the tumor [15]. Therefore, it is expected that increased fibrosis and degenerative changes in tumor cells due to neoadjuvant therapy in the fibroadipous parenchyma will make the differentiation of the tumor more difficult (Figure 5).

Even if a clear margin is obtained as a result of the intraoperative frozen evaluation, 10% false negativity is reported in the histopathological examination of the permanent tissue [16, 17]. Intraoperative surgical margin assessment in this histological subtype is performed with a small number of samples in narrow areas, which raises some doubts about the safety of its resection [18]. In the studies of Piper et al., the intraoperative positive margin rate in ILC is reported to be 37.6% [19].

In our study, surgical margins were positive in 26 (50%) of 52 cases in the frozen session. Cavity revision was performed in 8 patients (30.8%) who underwent additional surgery, and completion mastectomy was performed in 18 patients (69.2%). However, in 7 of 8 patients who underwent cavity revision and 5 of 18 patients who underwent completion mastectomy, no tumors were detected in the reoperation materials in the permanent sections. In our study, the rate of tumor absence in the reoperation materials was 46.1%, and a small tumor size was found to be associated with our series. This high rate may be due to the freezing artifacts formed in the tissue in the frozen session, the single tumor cells becoming more obscure, the confusion of tumor cells with inflammatory or stromal cells, and the complete deletion of cellular details at the surgical margins. Furthermore, it should be kept in mind that tumor sampling from the correct area may not be performed in the frozen session, since the absence of a stromal reaction in the tumor causes the invasive edges of the tumor to not be evaluated clearly. It was thought that the absence of tumor in the permanent sections in 5 of 18 cases who underwent complementary mastectomy may be due to false positives in the frozen evaluation, as well as sampling errors in the permanent tissue. Complete sampling and histopathological examination of the mastectomy material is not possible in terms of the functioning of the pathology laboratory, time, and cost. Therefore, since mastectomy specimens were not completely sampled, it was thought that the tumor might not have been detected in the permanent sections.

Regarding neoadjuvant therapy in invasive lobular carcinomas, many retrospective studies and meta-analyses report low pathologic complete response, high positive surgical margin, and low clinical response compared to ductal carcinoma, and neoadjuvant therapy is considered a relative contraindication [20–24]. The pathological complete response rate of neoadjuvant therapy varies between 1 and 7.4% in the literature [20, 22–24]. In our study, no pathological complete response was observed in any of the 12 patients who received neoadjuvant therapy in our population. However, in radiological response evaluation, 7 (58.3%) of 12 patients had a complete response and 3 (25%) had a partial response. Pathological diagnostic difficulties arising from the unique pattern of the tumor in ILC are also reported in its radiological diagnosis [25]. The difference in the evaluation of pathological and radiological responses in our series causes us to think that residual breast follow-up may be difficult in patients undergoing BCS, even if a safe margin is achieved surgically.

In our series, the need for additional surgery was not found to be statistically significant between the patients who received and did not receive neoadjuvant therapy, due to the low number of cases. BCS was initially applied to 7 (58.3%) of the patients who received neoadjuvant therapy. An additional surgical procedure was performed in 6 (85.7%) of 7 patients who underwent BCS, and 4 patients (57.1%) underwent a complementary mastectomy. Boughey et al., in their study, investigated the frequency of BCS in ILC patients who received and did not receive neoadjuvant therapy. In this study, BCS was 17% in those who received neoadjuvant therapy and 43% in those who did not, and concluded that neoadjuvant therapy did not increase the rate of BCS in ILC patients [24]. Tubiana Hulin et al. Reported the rate of BCS at 47% and completion mastectomy at 34% in patients with ILC who received neoadjuvant therapy [23]. Lips et al., in their study examining the radiological response evaluation of neoadjuvant therapy in ILC and invasive ductal carcinoma cases, response in ILCs was mostly multinodular and diffuse, and they found mass-like contrast in only 13% of the cases [26]. Reidel et al., on the other hand, reported that neoadjuvant therapy is a factor that increases false negativity in intraoperative surgical margin evaluation, especially in nonpalpable lesions [17].

In our series, in accordance with the literature, the need for additional surgery, which was detected at a high rate has been interpreted as evidence that changes in ILCs secondary to neoadjuvant therapy complicate intraoperative pathological assessment.

## 5. Conclusion

The importance of the pathological perspective in the success of the BCS procedure, which has been frequently preferred recently, is clear. We would like to emphasize that this type of breast cancer may not be evaluated with Hematoxylin-Eosin stained preparations in the evaluation of surgical margins in permanent tissues in pathology practice and immunohistochemical methods can be applied, therefore intraoperative evaluation may result in high failure rates. Freezing artifacts and neoadjuvant treatment effects are other factors that increase this failure.

ILC is now recognized as a discrete disease process, and growing clinical evidence, particularly in the era of personalized therapy, indicates that treatment strategies based on TNM classification for all invasive breast carcinomas, regardless of pathological features are not optimal for specific subtypes such as ILC.

The major limitation of our study is that it is a retrospective study, but our case series consists of a team of specialist surgeons, pathologists, radiologists, and oncologists, and differences in patient management are minimal. We think that randomized prospective clinical studies are needed to determine the best treatment strategies specific to this tumor subtype.

## Figures and Tables

**Table 1:** Clinical and biological characteristics of the cases.

Tumor characteristics	Cases
Age average	
Mastectomy	60,51 ± 13,64
BCS	58,94 ± 10,49

Molecular subtypes	
Luminal A	50
Luminal B	29

Multifocality	
Presence	37
Absent	42

In situ component	
Presence	50
Absent	29

T stage	
T1	27
T2	44
T3	8

Nodal stage	
N0	40
N1	20
N2	11
N3	8

Neoadjuvant treatment	
Presence	12
Absent	67

Surgical procedure applied	
Mastectomy	27
BCS	52
Completion mastectomy	18

**Table 2:** Clinicopathological features of patients who underwent BCS.

	No additional surgery	Additional surgery		Total
	Only lumpectomy	Cavity revision	Completion mastectomy	
Age				
Under 50	6	1	5	12
Over 50	20	7	13	40

Tumor size				
T1	13	5	4	22
T2	13	3	11	27
T3	0	0	3	3

Multifocality				
Presence	8	2	11	21
Absent	18	6	7	31

Neoadjuvant treatment				
Presence	1	2	4	7
Absent	25	6	14	45

In situ component				
Presence	15	5	13	33
Absent	11	3	5	19
Total	26	8	18	52

**Table 3:** Surgical procedures performed in patients who received and did not receive neoadjuvant.

Cases	Receiving neoadjuvant therapy	Not receiving neoadjuvant therapy	Total
Mastectomy	5	22	27
BCS	7	45	52
Additional surgery after BCS	6	20	26
Total	12	67	79

## Data Availability

The data used to support the findings of this study are included within the article.
